# Age as a significant prognostic factor in patients meeting the global ARDS definition but not the Berlin definition

**DOI:** 10.3389/fmed.2026.1929746

**Published:** 2026-10-12

**Authors:** Pan Pan, Jin Chen, Tiantian Zhang, Cheng Haibo, Zhang Wanyi, Xiaobo Han, Han Xinjie, Hu Xingshuo, Yang Qingyun, Gu Hongjun, Liu Yuhong, Lixin Xie

**Affiliations:** 1 Senior Department of Pulmonary and Critical Care Medicine, Chinese PLA General Hospital, Beijing, China; 2 Shandong Future Network Research Institute, Jinan, China; 3 Jiangsu Future Network Group Co, Nanjing, China; 4 Department of Respiratory and Critical Care Unit, The First Affiliated Hospital of Zhengzhou University, Zhengzhou, China

**Keywords:** age, ARDS, COVID-19, outcome, risk stratification

## Abstract

**Purpose:**

ARDS (Acute Respiratory Distress Syndrome) poses significant challenges in critical care, particularly during the COVID-19 pandemic. The new global definition of ARDS includes some patients who are not previously considered to have ARDS. This study aims to look at the prognostic characteristics of these patients and develop a prediction model.

**Methods:**

This multicenter retrospective study included 5,214 patients from the Chinese People's Liberation Army General Hospital between December 2022 and February 2023. Patients were categorized into four groups based on the 2023 Global Definition and 2012 Berlin Definition: Global-definition-only ARDS + COVID-19, Global-definition-only ARDS−COVID-19, Berlin ARDS + COVID-19, and Berlin ARDS−COVID-19. Patient characteristics were analyzed using LASSO regression and Cox proportional hazards models to identify factors associated with 28-day mortality. A prognostic model was developed using key variables.

**Results:**

Among ARDS patients, the Berlin ARDS + COVID-19 group had the highest mortality, while the observed 28-day mortality in the Global-definition-only ARDS + COVID-19 group was of a similar magnitude to that in the Berlin ARDS without COVID-19 group. LASSO selected seven candidate predictors: heart rate, lactate dehydrogenase, platelet count, total bilirubin, D-dimer, age, and antiviral therapy. In the multivariable Cox model, lactate dehydrogenase, age, and antiviral therapy were independently associated with 28-day mortality. Compared with patients aged <75 years, the HRs for 28-day mortality were 2.09 (95% CI 1.07–4.10) for those aged 75 to <85 years and 3.78 (95% CI 2.08–6.86) for those aged ≥85 years. Antiviral therapy was associated with a lower hazard of 28-day mortality (HR 0.58, 95% CI 0.40–0.84).The model's AUC for predicting 28-day mortality was 0.732 in the training set and 0.726 in the validation set.

**Conclusions:**

The 2023 Global ARDS definition holds significant value in identifying COVID-19 patients receiving high-flow nasal oxygen (HFNO), the Global definition may facilitate earlier identification of ARDS patients. Age is a critical risk factor, highlighting the need for heightened clinical attention in elderly patients. Antiviral therapy was associated with a lower hazard of 28-day mortality. Seven candidate predictors were selected by LASSO and evaluated in a Cox prognostic model for 28-day mortality.

## Introduction

During World War II, medical workers noticed that patients with head, chest, and abdominal injuries were prone to developing “wet lungs.” These patients often had lower resuscitation success rates compared to those with “dry lungs” (1). It wasn't until 1967 that Ashbaugh et al. published a landmark paper describing Acute Respiratory Distress Syndrome (ARDS) as a syndrome characterized by pulmonary edema, atelectasis, interstitial and alveolar edema, and hyaline membrane formation. Clinically, it presents with rapid breathing, refractory hypoxemia, decreased compliance, and bilateral lung infiltrates, with a mortality rate of up to 58%. Patients receiving positive end-expiratory pressure (PEEP) treatment seemed to have better outcomes (2). Since then, attempts have been made to better standardize the definition of ARDS. However, neither the early AECC consensus definition nor the 2012 Berlin definition could provide a gold standard for diagnosing ARDS, only assessing the risk of ARDS in patients who meet the criteria (3). The main reason is that ARDS is a syndrome, not a disease, meaning it can have various causes and pathophysiological mechanisms. Studies have shown that diffuse alveolar damage (DAD) is not present in all ARDS patients (4). This has led clinical experts to rethink the definition and diagnosis of ARDS, with some studies suggesting the concept of ARDS mimics (5).

Despite significant progress with the 2012 Berlin definition, many practical issues remain. First, there is high heterogeneity in epidemiology and clinical outcomes across different healthcare settings. The incidence of ARDS varies widely, from 1.5 to 79 cases per 100,000 population, with lower rates reported in European countries compared to the U.S. Additionally, studies from Brazil report incidence rates ranging from 1.8 to 31 cases per 100,000 (6). These differences suggest varying perceptions of whether patients meet ARDS diagnostic criteria. The SAFE study, the largest international cohort study of ARDS patients, aimed to assess whether clinicians could quickly identify and diagnose ARDS patients in clinical practice. It found significant delays or missed diagnoses, with two-thirds of ARDS diagnoses delayed or missed and a 40% overall missed diagnosis rate. Recognition rates for mild ARDS were 51% and 79% for severe ARDS (7). The ARDS definition also has limitations in resource-poor settings. For example, arterial blood gas, ventilators, and chest imaging required by the Berlin definition are often unavailable in less developed areas, making ARDS diagnosis challenging (8). Moreover, many RCTs targeting ARDS patients have yielded negative results, often showing no significant benefit or mortality difference in the intervention groups, complicating treatment studies (9).

The global COVID-19 pandemic highlighted the limitations of the ARDS definition, with many studies indicating difficulties in diagnosing COVID-19 patients with ARDS using the current criteria. COVID-19 patients often have less endothelial damage and exudate, with milder clinical presentations not matching the severity seen in laboratory and imaging results. COVID-19-related ARDS has an onset time of 8–12 days, inconsistent with the Berlin definition's one-week onset criterion. Additionally, HFNO may be safe and effective for moderate to severe COVID-19-related ARDS patients. These discrepancies necessitate a reevaluation of the ARDS definition.

In this context, the 2023 Global definition introduced changes to make ARDS diagnosis more feasible, particularly in resource-limited areas, such as using SpO2/FiO2 and various imaging methods. The most fundamental change allows patients receiving HFNO >30 L/min to be included in the diagnostic criteria. Some scholars believe this broadened definition could reduce specificity, especially for such a heterogeneous syndrome, potentially increasing false-positive ARDS cases. This study uses data from patients during the COVID-19 pandemic in mainland China to compare groups based on the new and Berlin definitions, verify the significance of the Global-definition-only ARDS, identify prognostic factors, and determine the scope and application of the Global-definition-only ARDS to better guide clinical ARDS diagnosis and treatment.

## Methods

### Participants

Patients were selected from the First, Third, Fourth, Sixth, Seventh, and Eighth Medical Centers of the Chinese People's Liberation Army General Hospital between December 10, 2022, and February 20, 2023. Patients who fulfilled the Berlin Definition were classified as the Berlin ARDS group, whereas patients who fulfilled the 2023 Global ARDS Definition but did not meet the Berlin Definition were classified as the Global-definition-only ARDS group. In this cohort, the principal distinguishing respiratory-support criterion for the Global-definition-only group was the use of HFNO at a flow rate ≥30 L/min rather than positive-pressure ventilation with PEEP ≥5 cmH_2_O.

Exclusion criteria included: (1) patients younger than 18 years; (2) hospital stay less than 24 h; (3) death within 24 h of admission. Patients who died or were discharged within 24 h were excluded because complete baseline data required for ARDS classification and prognostic modeling were not consistently available within this early period.Patients were first divided into COVID-19 positive and negative groups. Each group was then subdivided into Berlin ARDS and Global-definition-only ARDS groups. Patients were categorized into four groups: Berlin ARDS + COVID-19, Berlin ARDS—COVID-19, Global-definition-only ARDS + COVID-19, and Global-definition-only ARDS—COVID-19, and analyzed further. This retrospective cohort study was approved by the Ethics Committee of the Chinese PLA General Hospital (approval No. 309202302230712).

### LASSO analysis for feature selection

To explore prognostic factors associated with 28-day mortality among patients who met the 2023 Global ARDS Definition but not the Berlin Definition (*n* = 458), we employed LASSO regression as a variable-screening tool. LASSO applies an L1 penalty function that shrinks regression coefficients toward zero, effectively removing variables with minimal predictive contribution and retaining those most relevant to the outcome. To ensure comparability across different units and scales, all continuous predictors were standardized prior to analysis. The optimal penalty parameter *λ* was determined via 10-fold cross-validation based on the minimum deviance criterion. A total of 48 clinical and laboratory variables were included as candidate predictors, with 28-day mortality as the dependent variable. Missing data were handled using multiple imputation by chained equations (MICE).

### Cox proportional hazards model

To evaluate the independent prognostic value of the LASSO-retained variables, we constructed a multivariable Cox proportional hazards model. Patients meeting the Global definition of ARDS were randomly divided into a training set (70%) and a validation set (30%), with stratification by COVID-19 status to ensure balanced distribution. Age was categorized as <75 years, 75 to <85 years, and ≥85 years, with <75 years as the reference category. Antiviral therapy was defined as treatment with nirmatrelvir/ritonavir (Paxlovid) or Azvudine. Variables that remained statistically significant (*P* < 0.05) in the Cox model were considered independent predictors of 28-day mortality. Results are presented as hazard ratios (HRs) with 95% confidence intervals (CIs).

### Statistical analysis

Continuous variables were described as mean ± standard deviation or median (P25, P75), and categorical variables as count (percentage). Group comparisons were conducted using variance analysis or non-parametric tests for continuous variables, and chi-square tests for categorical variables. Survival rates were evaluated using Kaplan–Meier curves and log-rank tests. Areas under receiver operating characteristic curves were used to evaluate how well the model distinguished survivors and non-survivors. Statistical analyses were performed with R software version 3.5.0, with a *p*-value less than 0.05 considered significant.

## Result

### Patient characteristics

Among the 5,214 included patients, 928 met the 2023 Global ARDS Definition. Of these, 470 also met the Berlin Definition, while 458 met the Global Definition but not the Berlin Definition. The remaining 4,286 patients did not meet the Global ARDS criteria (Table 1). Figure 1 shows flowchart of the patients involved in this study.

**Table 1:** The baseline of the patients involved in this study.

Characteristic	Category	Non-ARDS (*n* = 4,286)	Global-definition-only ARDS (*n* = 458)	Berlin ARDS (*n* = 470)	*P* value
Age, years	<75	2,671 (62.3)	113 (24.7)	171 (36.4)	<0.001
	75 to <85	737 (17.2)	115 (25.1)	120 (25.5)	
	≥85	878 (20.5)	230 (50.2)	179 (38.1)	
Gender	Female	1,779 (41.5)	146 (31.9)	122 (26.0)	<0.001
	Male	2,507 (58.5)	312 (68.1)	348 (74.0)	
Smoking history	No	3,722 (91.6)	415 (93.9)	389 (91.5)	0.235
	Yes	343 (8.4)	27 (6.1)	36 (8.5)	
Days from symptom onset, days		8.27 (8.14)	8.70 (6.28)	8.68 (7.23)	0.358
Body temperature, °C		36.78 (0.96)	36.88 (0.73)	36.77 (1.93)	0.161
Heart rate, beats/min		81.37 (12.86)	84.79 (16.29)	89.86 (18.62)	<0.001
Systolic blood pressure, mmHg		127.60 (18.13)	131.52 (20.22)	132.51 (23.35)	<0.001
Diastolic blood pressure, mmHg		76.57 (23.41)	74.04 (12.28)	73.66 (13.92)	0.004
Lactate dehydrogenase, U/L		239.59 (195.58)	318.11 (185.96)	423.94 (448.47)	<0.001
Creatine kinase, U/L		67.00 [41.00, 114.00]	76.00 [39.75, 180.15]	112.90 [49.30, 289.40]	<0.001
Procalcitonin, ng/mL		0.08 [0.05, 0.50]	0.50 [0.09, 0.70]	0.50 [0.20, 2.16]	<0.001
Total cholesterol, mmol/L		4.00 (1.24)	3.58 (1.12)	3.51 (1.22)	<0.001
White blood cell count, ×10^9^ /L		5.74 [4.26, 7.84]	7.80 [5.31, 10.98]	9.35 [6.74, 13.97]	<0.001
Lymphocyte count, ×10^9^ /L		1.02 [0.64, 1.51]	0.63 [0.40, 0.95]	0.58 [0.35, 0.90]	<0.001
Platelet count, ×10^9^ /L		200.67 (88.86)	179.03 (82.47)	169.53 (88.07)	<0.001
Hemoglobin, g/L		121.99 (25.68)	113.28 (29.61)	108.42 (32.61)	<0.001
Albumin, g/L		35.96 (6.24)	31.11 (4.64)	30.45 (5.36)	<0.001
Alanine aminotransferase, U/L		20.00 [13.00, 31.60]	22.30 [14.20, 36.00]	24.35 [15.78, 44.47]	<0.001
Aspartate aminotransferase, U/L		26.10 [19.40, 37.00]	32.30 [23.15, 51.80]	36.15 [23.72, 64.53]	<0.001
Total bilirubin, μmol/L		9.90 [7.30, 13.70]	10.50 [7.20, 14.95]	11.30 [7.50, 16.60]	<0.001
Creatinine, μmol/L		72.00 [57.50, 90.40]	85.10 [66.70, 117.50]	97.60 [67.47, 164.12]	<0.001
Blood urea nitrogen, mmol/L		7.40 (6.60)	11.67 (9.56)	15.79 (12.40)	<0.001
D-dimer, mg/L		0.41 [0.20, 1.09]	0.92 [0.44, 2.81]	2.07 [0.76, 6.66]	<0.001
C-reactive protein, mg/L		17.00 [4.00, 51.92]	68.09 [30.79, 117.80]	83.39 [39.97, 144.16]	<0.001
Interleukin-6, pg/mL		12.50 [4.68, 39.71]	42.91 [16.62, 127.25]	86.38 [23.87, 261.78]	<0.001
Red blood cell count, ×10^9⁹/L		4.15 (0.77)	3.97 (0.85)	3.98 (0.85)	<0.001
Neutrophil count, ×10^9⁹/L		3.04 [1.30, 4.95]	5.30 [2.78, 8.92]	4.58 [0.92, 9.07]	<0.001
Blood glucose, mmol/L		7.38 (4.74)	9.46 (4.39)	10.86 (6.03)	<0.001
Cardiovascular disease	No	3,126 (72.9)	208 (45.4)	265 (56.4)	<0.001
	Yes	1,160 (27.1)	250 (54.6)	205 (43.6)	
Cerebrovascular disease	No	3,572 (83.3)	302 (65.9)	301 (64.0)	<0.001
	Yes	714 (16.7)	156 (34.1)	169 (36.0)	
Chronic respiratory disease	No	3,814 (89.0)	359 (78.4)	401 (85.3)	<0.001
	Yes	472 (11.0)	99 (21.6)	69 (14.7)	
Rheumatoid arthritis or connective tissue disease	No	4,159 (97.0)	442 (96.5)	452 (96.2)	0.512
	Yes	127 (3.0)	16 (3.5)	18 (3.8)	
Gastrointestinal disease	No	3,635 (84.8)	362 (79.0)	359 (76.4)	<0.001
	Yes	651 (15.2)	96 (21.0)	111 (23.6)	
Diabetes mellitus	No	3,221 (75.2)	288 (62.9)	300 (63.8)	<0.001
	Yes	1,065 (24.8)	170 (37.1)	170 (36.2)	
Liver disease	No	3,746 (87.4)	341 (74.5)	315 (67.0)	<0.001
	Yes	540 (12.6)	117 (25.5)	155 (33.0)	
Malignancy	No	3,503 (81.7)	355 (77.5)	377 (80.2)	0.075
	Yes	783 (18.3)	103 (22.5)	93 (19.8)	
Hypertension	No	2,428 (56.6)	180 (39.3)	192 (40.9)	<0.001
	Yes	1,858 (43.4)	278 (60.7)	278 (59.1)	
Hematological disease	No	4,108 (95.8)	443 (96.7)	452 (96.2)	0.643
	Yes	178 (4.2)	15 (3.3)	18 (3.8)	
History of transplantation	No	4,148 (96.8)	444 (96.9)	454 (96.6)	0.956
	Yes	138 (3.2)	14 (3.1)	16 (3.4)	
Glucocorticoids use	No	3,023 (70.5)	170 (37.1)	192 (40.9)	<0.001
	Yes	1,263 (29.5)	288 (62.9)	278 (59.1)	
Invasive positive-pressure ventilation	No	4,274 (99.7)	458 (100.0)	75 (16.0)	<0.001
	Yes	12 (0.3)	0 (0.0)	395 (84.0)	
Nintedanib use	No	4,285 (100.0)	449 (98.0)	460 (97.9)	<0.001
	Yes	1 (0.0)	9 (2.0)	10 (2.1)	
Tocilizumab use	No	4,236 (98.8)	413 (90.2)	402 (85.5)	<0.001
	Yes	50 (1.2)	45 (9.8)	68 (14.5)	
Baricitinib use	No	4,240 (98.9)	439 (95.9)	448 (95.3)	<0.001
	Yes	46 (1.1)	19 (4.1)	22 (4.7)	
COVID-19 status	Negative	952 (22.2)	33 (7.2)	65 (13.8)	<0.001
	Positive	3,334 (77.8)	425 (92.8)	405 (86.2)	
28-day outcome	Alive	4,108 (95.8)	282 (61.6)	176 (37.4)	<0.001
	Dead	178 (4.2)	176 (38.4)	294 (62.6)	
28-day survival time, days		27.19 (4.07)	20.98 (9.80)	17.14 (9.87)	<0.001

Values are presented as *n* (%), mean (SD), or median [IQR], as appropriate.

Global-definition-only ARDS denotes patients who met the 2023 Global ARDS Definition but did not meet the Berlin Definition.

COVID-19 status in the non-ARDS group was corrected to 3,334 positive and 952 negative, consistent with Figure 1.

**Figure 1:**
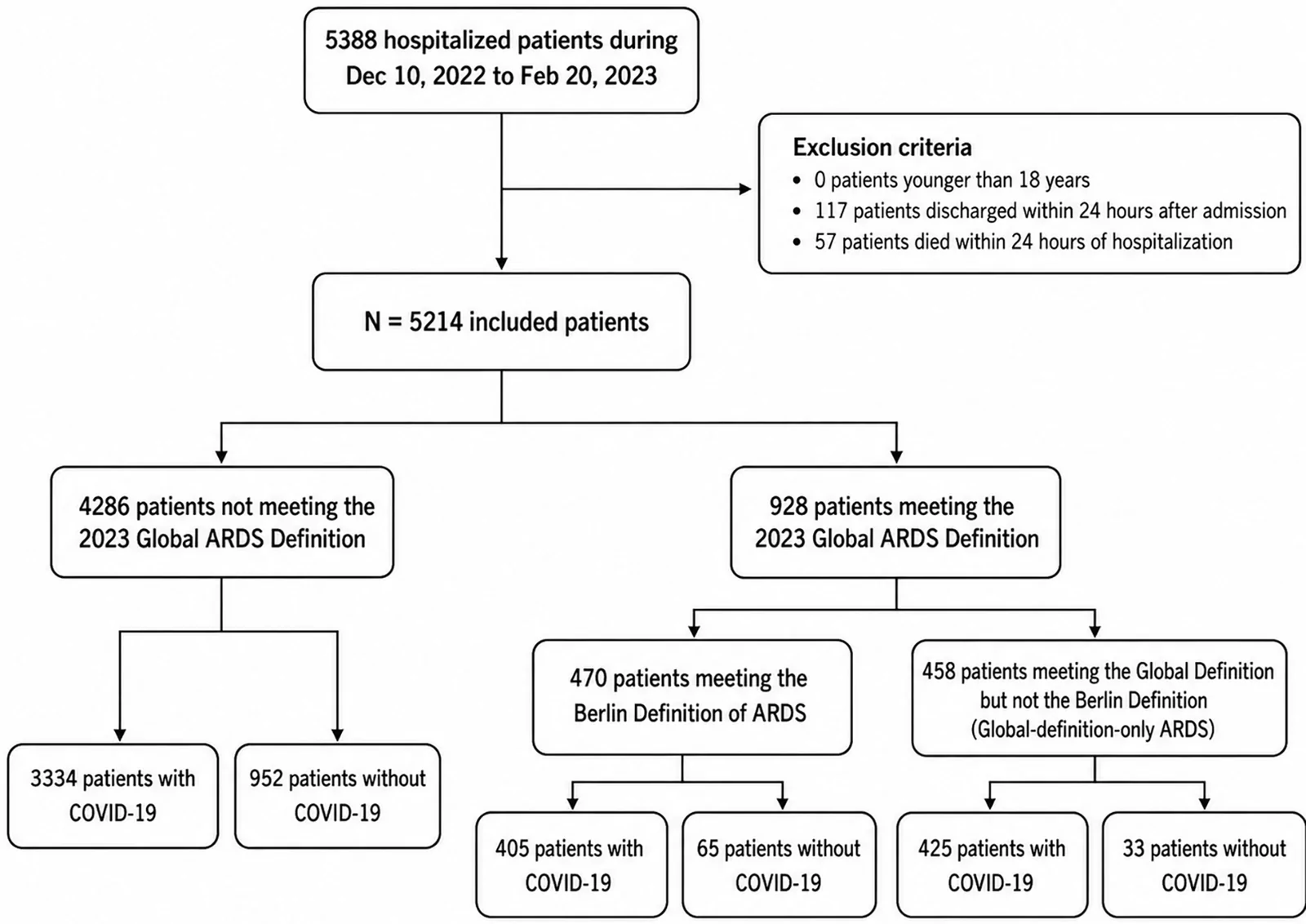
Flowchart of the patients involved in this study.

### Survival analysis of different groups

Figure 2 compares survival rates among different groups. Non-ARDS patients had the lowest mortality. Among ARDS patients, the Berlin ARDS + COVID-19 group had the highest observed mortality, whereas the observed mortality in the Global-definition-only ARDS + COVID-19 group was numerically close to that in the Berlin ARDS without COVID-19 group.

**Figure 2:**
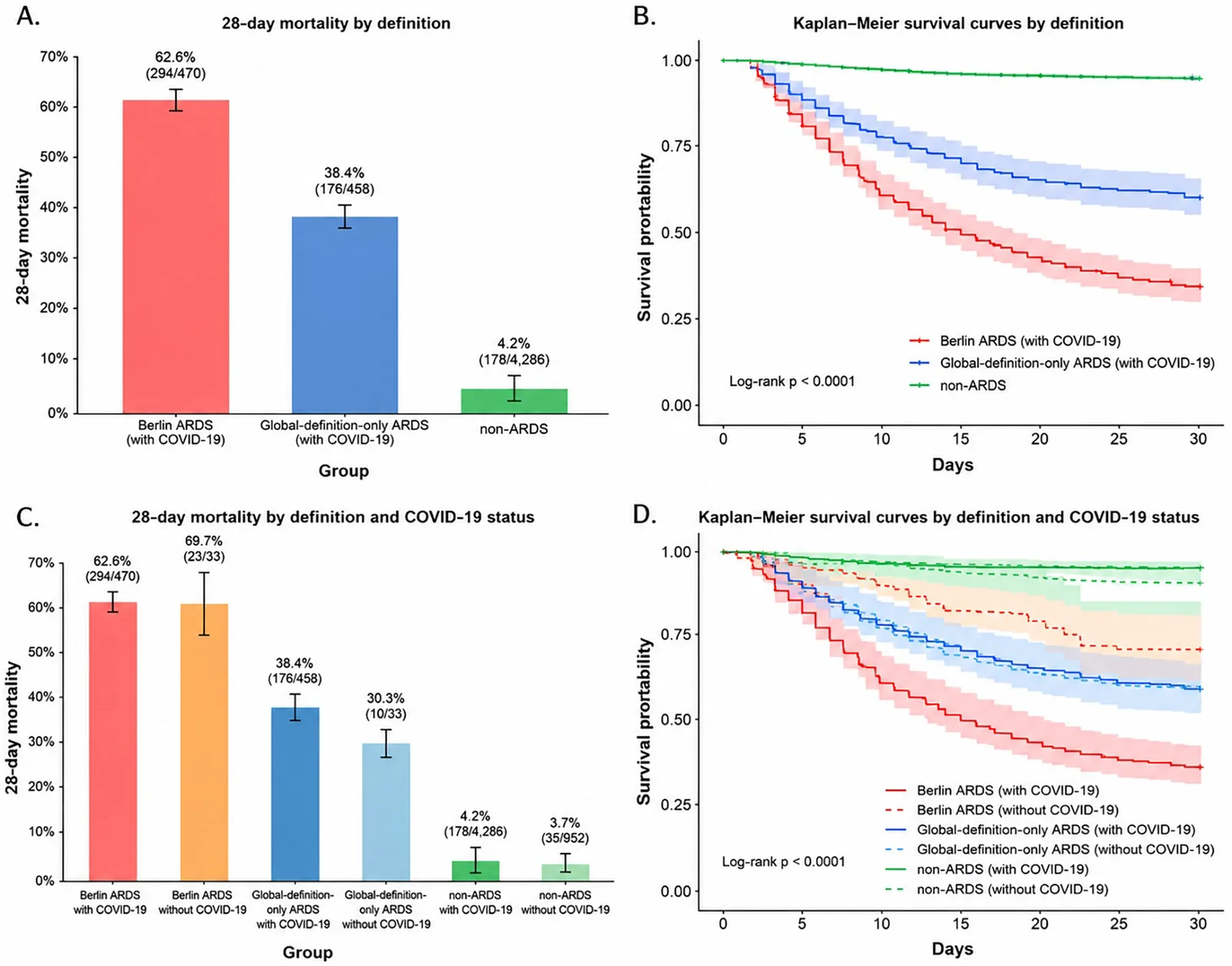
Twenty-eight-day mortality and survival curves in different patient groups. Panels **A** and **C** show 28-day mortality proportions; panels **B** and **D** show Kaplan–Meier survival curves.

### Key features of mortality in global-definition-only ARDS patients

To identify prognostic factors associated with 28-day mortality among patients newly included by the Global ARDS Definition, LASSO regression was performed in the 458 Global-definition-only ARDS patients. LASSO regression retained seven candidate predictors: heart rate, lactate dehydrogenase, platelet count, total bilirubin, D-dimer, age, and antiviral therapy. The age showed the largest absolute standardized coefficient among the variables retained by the LASSO model, indicating the highest relative importance for predicting 28-day mortality (Figure 3).

**Figure 3:**
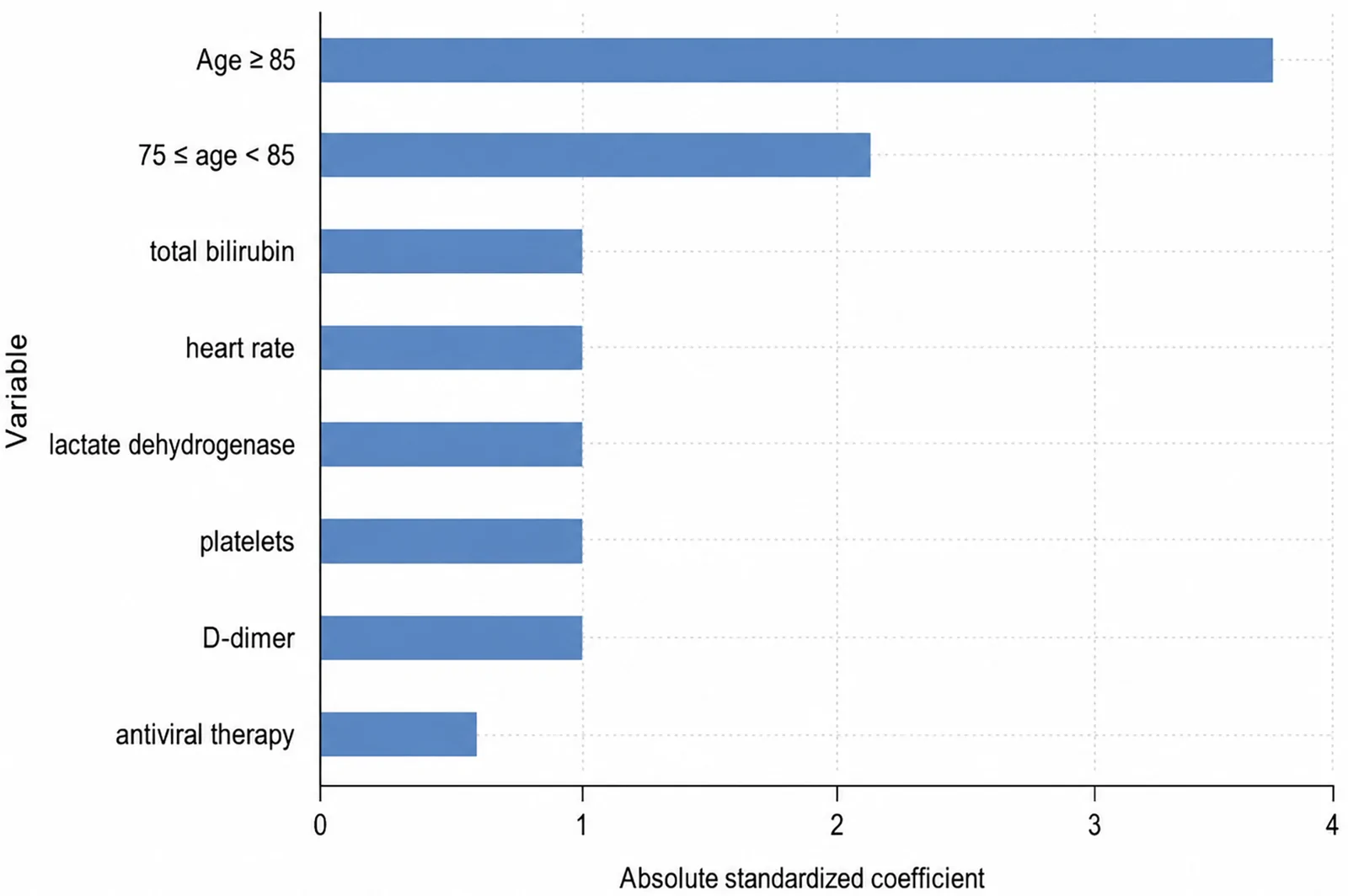
Absolute standardized coefficients of variables retained by the LASSO model for predicting 28-day mortality among patients meeting the global but not the Berlin ARDS definition.

### Cox regression analysis of mortality risk factors

In the multivariable Cox model, lactate dehydrogenase, age, and antiviral therapy were independently associated with 28-day mortality, whereas heart rate, platelet count, total bilirubin, and D-dimer were not statistically significant.

Compared with patients aged <75 years, those aged 75 to <85 years had a 109% higher hazard of 28-day mortality (HR 2.09, 95% CI 1.07–4.10), whereas those aged ≥85 years had a 278% higher hazard (HR 3.78, 95% CI 2.08–6.86). Antiviral therapy was associated with a lower hazard of 28-day mortality (HR 0.58, 95% CI 0.40–0.84). These results are shown in Table 2.

**Table 2:** Multivariable Cox regression analysis of factors associated with 28-day mortality in patients with Global-definition-only ARDS.

Variable	HR (95% CI)	*P* value
Heart rate	1.010 (0.999–1.021)	0.069
Lactate dehydrogenase	**1.001** (**1.001–1.002)**	**<0**.**001**
Platelet count	0.998 (0.996–1.001)	0.233
Total bilirubin	1.013 (0.997–1.029)	0.109
D-dimer	0.995 (0.976–1.014)	0.585
Age 75 to <85 vs <75 years	**2.092** (**1.068–4.099)**	**0**.**032**
Age ≥85 vs <75 years	**3.778** (**2.079–6.864)**	**<0**.**001**
Antiviral therapy	**0.579** (**0.398–0.843)**	**0**.**004**

Bold values indicate statistical significance (*P* < 0.05).

### Prognostic model performance

Figure 4 presents the time-dependent ROC curves of the prediction model in the training and validation cohorts. In the training cohort, the AUCs for predicting 7-, 14-, and 28-day mortality were 0.704, 0.721, and 0.732, respectively. In the validation cohort, the corresponding AUCs were 0.723, 0.722, and 0.726. The comparable performance between the training and validation cohorts suggests that the model achieved stable discrimination and good generalizability for predicting short-term mortality.

**Figure 4:**
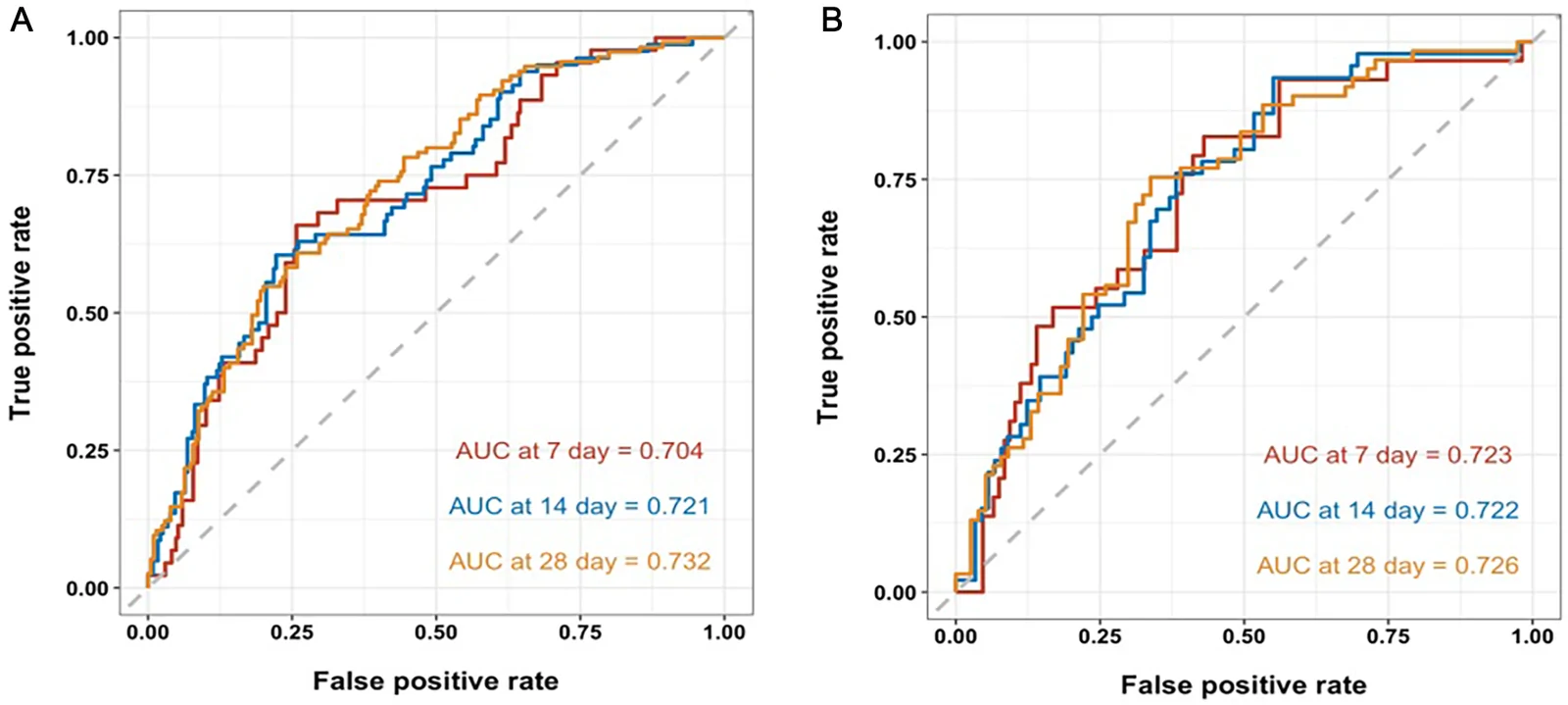
Time-dependent ROC curves of the prediction model for 7-, 14-, and 28-day mortality. **(A)** Training cohort. **(B)** Validation cohort. In the training cohort, the AUCs at 7, 14, and 28 days were 0.704, 0.721, and 0.732, respectively. In the validation cohort, the corresponding AUCs were 0.723, 0.722, and 0.726.

## Discussions

The 2023 Global ARDS definition includes non-mechanically ventilated patients with severe hypoxemia. The observed 28-day mortality in COVID-19 patients meeting the Global-definition-only ARDS criteria was numerically close to that observed in non-COVID-19 patients meeting the Berlin criteria.Age was the most significant risk factor, with antiviral therapy being protective. We identified seven indicators related to mortality and developed a predictive model for 28-day mortality in this patient group.

Firstly, our study found that the mortality rate of patients who met the Berlin definition and had COVID-19 was higher than that of patients who met the Global-definition-only ARDS criteria and had COVID-19.This finding is consistent with lower observed mortality among COVID-19 patients classified by the Global-definition-only criteria, many of whom were managed with HFNO, compared with those meeting the Berlin definition. According to the Berlin definition, patients using HFNO are not considered ARDS patients, but they should not be excluded. Our study found that the mortality rate in this group of patients could still reach 40%. In a multicenter observational study of patients with COVID-19-associated acute hypoxemic respiratory failure, ICU mortality was lower among patients initially treated with HFNO than among those receiving invasive ventilation (22.7% vs. 35.6%, *P* = 0.001) (11). Other research has found that almost all patients with bilateral pulmonary infiltrates and a PaO2/FiO2 ratio of 300 mm Hg meet the oxygenation and chest x-ray criteria for ARDS but do not meet the Berlin criteria for positive pressure ventilation (10). This indicates that the severity of hypoxemia is more important than the mode of respiratory support, and the mode of respiratory support does not represent the patient's true respiratory status. Moreover, during the COVID-19 outbreak, we must acknowledge that the unprecedented demand for ICUs may lead to clinical decision changes due to resource limitations (11). Even in non-COVID-19 situations, when a patient's oxygen demand is still increasing, we need to be aware of whether early lung injury has occurred, and intubation and mechanical ventilation are just treatment measures in the gradual progression of respiratory failure. Different physicians may choose different times for intubation, but this practical difference should not be considered a difference in ARDS diagnosis (12). Some studies have reported that non-ventilated patients who meet other ARDS diagnostic criteria have similar characteristics and outcomes to ARDS patients. For example, hypoxemic patients with bilateral infiltrates treated with HFNO show a similar pattern of plasma inflammatory and injury biomarkers to ARDS patients receiving mechanical ventilation (13). Therefore, expanding the diagnosis of ARDS may help in the early diagnosis of ARDS in HFNO patients, allowing for early ARDS intervention (14), which can significantly improve patient outcomes (15). This can be said to be the intention behind the new ARDS definition, aiming to identify these patients and provide timely treatment. Whether such earlier recognition leads to changes in management or improved clinical outcomes requires evaluation in prospective interventional studies.

Our study results suggest that age is the most significant risk factor in the Global-definition-only ARDS with COVID-19 group. In fact, the high mortality rate of elderly COVID-19 patients has been a major issue throughout the entire COVID-19 pandemic. According to the World Health Organization, the overall mortality rate of COVID-19 is estimated to be about 2%, but the mortality rate for elderly patients rises sharply, with those over 80 years old having an estimated mortality rate as high as 15%–20% [World Health Organization. Coronavirus (COVID-19) dashboard, https://covid19.who.int/]. Additionally, there are almost no elderly patients who survive after being placed on ECMO (16).

Considering the reasons, elderly patients are more likely to have underlying health conditions such as heart disease, diabetes, and respiratory diseases, which can exacerbate the severity of COVID-19 symptoms. Cilloniz et al. reported an observational study of critically ill COVID-19 patients in 55 ICUs in Spain. In this analysis, she studied the mortality risk factors of 5090 mechanically ventilated patients, among whom 1,525 (27%) were over 70 years old. The overall hospital mortality rate for patients over 70 was twice that of those under 70 (50% vs. 23%). Factors independently associated with increased hospital mortality for patients over 70, besides age, included ventilation status at ICU admission, chronic heart disease, chronic renal failure, platelet count, and recent hospitalization within the past 30 days. The platelet count aligns with our study's results (17).

The PRoVENT-COVID study attempted to clarify the mechanical ventilation parameters in patients of different age groups and found no significant differences in ventilation parameters and rescue treatments on the first day of ventilation. However, elderly patients had a higher frequency of tracheostomy, were more prone to acute kidney injury and myocardial infarction, had longer ICU and hospital stays, and had higher mortality rates (16.6 vs. 27.7 vs. 44.3 vs. 55.6%) (18, 19). Some studies have found that major risk factors for severe COVID-19 include age, male sex, obesity, smoking, hypertension, type 2 diabetes mellitus, and other comorbid chronic conditions (19–21).In addition to the high incidence of comorbidities among the elderly, age-related impairment of immune defense against SARS-CoV-2 infection, or immunosenescence, and increased risk for immunopathology also play a major role in contributing to heightened vulnerability to severe COVID-19 outcomes in older adults. Researchers speculate that cytokine storms and immunopathology secondary to inflammation and immunosenescence are the reasons for the complex changes in immune mechanisms. Treatments targeting these mechanisms, such as anti-IL-6 therapy and the efficacy of COVID-19 vaccines, may precisely intervene at these points.

In addition, our research has found that antiviral therapy was associated with a lower hazard of 28-day mortality. The antiviral drugs we used include Paxlovid or Azvudine. It's important to note that here we define patients receiving antiviral therapy as those with mild to moderate COVID-19 and risk factors for severe disease who are within 5 days of onset, or patients who are beyond 5 days of onset but have a viral nucleic acid CT value of less than 30. Many studies have addressed the effectiveness of antiviral therapy. One study involved participants being randomly assigned to receive either Paxlovid or a placebo for 5 consecutive days. This study showed that among the participants who received Paxlovid treatment within 3 days of symptom onset, only 3 out of 339 participants (0.8%) were still hospitalized on day 28, with no reported deaths. In contrast, 7% (27/385) of patients receiving placebo treatment were still hospitalized on day 28, with 7 deaths reported (*p* < 0.0001). Among the participants who received treatment within 5 days of symptom onset, 1% (6/607) of those who received Paxlovid treatment were still hospitalized on day 28, while the placebo group had 6.7% (41/612) of patients still hospitalized. As of day 28, there were no reported deaths in the Paxlovid group, while the placebo group had 10 deaths (1.6%) (22). Zheng et al. conducted a meta-analysis on the effectiveness and safety of Paxlovid in COVID-19, including 186,306 patients from 13 studies. The results showed that the mortality rate and length of hospital stay in the Paxlovid group were significantly lower than those in the control group [OR 0.22 (95% CI, 0.11–0.45; *I*^2^ = 93%), *P* < 0.0001], while the probability of adverse events and rebound occurrence in patients showed no statistical difference (OR 1.07; 95% CI, 0.49–2.34; *I*^2^ = 90%, *P* = 0.87) (23). Chinese guidelines allow the use of Azvudine and Paxlovid in COVID-19 patients, especially those with pre-existing comorbidities. Azvudine (FNC) is a nucleoside analog that inhibits HIV-1 RNA-dependent RNA polymerase (RdRp). Several studies have shown that Azvudine can reduce the duration of virus clearance and improve the clinical prognosis in COVID-19 patients, including those with pre-existing comorbidities (24) There are even studies suggesting that Azvudine may be more effective than Paxlovid in treating hospitalized COVID-19 patients with pre-existing comorbidities (25).

Of course, our research also has several limitations. Firstly, despite being a multicenter study, it was conducted during the unique period of the COVID-19 pandemic. Therefore, we lack relevant data to support conclusions regarding ARDS outside of COVID-19. Secondly, the exclusion of patients who died within 24 h may have introduced survivor-selection bias and may have underestimated early mortality; therefore, the prognostic findings should be interpreted in the context of patients surviving beyond the first 24 h of hospitalization. Thirdly, we did not stratify our study of elderly individuals by age to investigate the risk factors for severe ARDS among different age groups of elderly people. It remains unclear what disease backgrounds may lead to severe ARDS in this population. Antiviral therapy is a non-specific treatment method, and while our study suggests its early effectiveness for COVID-19 patients, the significance of the Global-definition-only ARDS in this context is noteworthy. In the post-pandemic era, with the immense pressure of new influenza infections, whether this Global-definition-only ARDS will meet our expectations requires further clinical research to provide answers. Lastly, given the relatively limited sample size and the large number of candidate variables initially considered, overfitting and instability of variable selection cannot be excluded; therefore, the selected predictors and model performance require validation in independent cohorts.

## Conclusion

The 2023 Global ARDS Definition identified an additional group of patients receiving HFNO who did not meet the Berlin criteria, predominantly in the setting of COVID-19. Among patients with Global-definition-only ARDS, older age was associated with a higher hazard of 28-day mortality, whereas antiviral therapy was associated with a lower hazard. Seven candidate predictors were selected by LASSO and evaluated in a Cox prognostic model. Given the retrospective design, the small non-COVID subgroup, and the lack of external validation, the broader applicability of these findings requires confirmation in prospective studies.

## Data Availability

The original contributions presented in the study are included in the article/Supplementary Material, further inquiries can be directed to the corresponding author.
